# Three-dimensional analysis of palatal morphology and PAS in patients with cleft lip and palate prior to orthodontic treatment

**DOI:** 10.1186/s13005-024-00440-2

**Published:** 2024-08-01

**Authors:** Maike Tabellion, Jörg Alexander Lisson

**Affiliations:** Department of Orthodontics (G56), Saarland University, Kirrberger Strasse 100, 66424 Homburg/Saar, Germany

**Keywords:** Palatal morphology, Palatal volume, Cleft lip and palate, Posterior airway space, Nasopharynx

## Abstract

**Background:**

Since many different conclusions of craniofacial anomalies and their relation to the posterior airway space coexist, this comparative clinical study investigated the palatal morphology concerning volumetric size, posterior airway space dimension and the adenoids of patients with and without a cleft before orthodontic treatment.

**Methods:**

Three-dimensional intraoral scans and cephalometric radiographs of *n* = 38 patients were used for data acquisition. The patients were divided into three groups: unilateral cleft lip and palate (*n* = 15, 4 female, 11 male; mean age 8.57 ± 1.79 years), bilateral cleft lip and palate (*n* = 8, 0 female, 8 male; mean age 8.46 ± 1.37 years) and non-cleft control (*n* = 15, 7 female, 8 male; mean age 9.03 ± 1.02 years). The evaluation included established procedures for measurements of the palatal morphology and posterior airway space. Statistics included Shapiro-Wilk-Test and simple ANOVA (Bonferroni) for the three-dimensional intraoral scans and cephalometric radiographs. The level of significance was set at *p* < 0.05.

**Results:**

The palatal volume and cephalometric analysis showed differences between the three groups. The palatal volume, the superior posterior face height and the depth of the bony nasopharynx of patients with cleft lip and palate were significantly smaller than for non-cleft control patients. The superior posterior face height of bilateral cleft lip and palate patients was significantly smaller than in unilateral cleft lip and palate patients (BCLP: 35.50 ± 2.08 mm; UCLP: 36.04 ± 2.95 mm; *p* < 0.001). The percentage of the adenoids in relation to the entire nasopharynx and the angle NL/SN were significantly bigger in patients with cleft lip and palate than in the non-cleft control. In particular, the palatal volume was 32.43% smaller in patients with unilateral cleft lip and palate and 48.69% smaller in patients with bilateral cleft lip and palate compared to the non-cleft control.

**Conclusions:**

Skeletal anomalies relate to the dimension of the posterior airway space. There were differences among the subjects with cleft lip and palate and these without a cleft. This study showed that the morphology of the palate and especially transverse deficiency of the maxilla resulting in smaller palatal volume relates to the posterior airway space. Even the adenoids seem to be affected, especially for cleft lip and palate patients.

## Introduction

Cleft lip and palate are the most common malformation in oral and maxillofacial region with an incidence of about 1 out of 500–1000 live births occurring with or without various syndromes. The incidence for Asian and Native American populations is 1 in 500 and for Europeans 1 in 1000 [6, 15]. 70% of cleft lip and palate patients are non-syndromic and without any further cognitive or craniofacial anomaly [6]. Development of the lip and palate starts during the fourth week of intrauterine life as maxillary and nasal processes fuse. Due to different genetic and environmental reasons, fusion can be incomplete or non-occurring resulting in cleft lip and/or palate [10, 25]. Clefts involving the lip occur with a 2:1 male to female ratio. The ratio for isolated clefts of the palate is 1:2 female to male. Unilateral cleft lip and palate ratio is 2:1 for cleft on the left side to cleft on the right side [6]. Cleft lip and palate patients may have problems with feeding, hearing, speaking and social acceptance. Their rehabilitation is challenging to healthcare professionals and requires surgery, orthodontic treatment, speech therapy and if necessary psychosocial intervention [2, 6, 10, 15, 25, 26]. Due to surgical procedures and resultant scar tissue, maxillofacial growth is restricted. Patients with cleft lip and palate show especially sagittal and transverse restrictions of the maxilla, resulting in maxillary micro- and retrognathia and crossbites [18, 21]. Growth inhibition has been investigated concerning timing and protocol of the surgical intervention and skills of the surgeons. 12 to 24 months of age are described as ideal timing for palatal cleft surgery [3, 21, 23]. Treatment starts immediately after birth and continues up to adulthood. Dimension and morphology of upper arches have been often investigated. Dental casts were measured for intermolar and intercanine distance, palatal length and depth [1, 9, 22, 23]. Compared to two-dimensional linear measurements three-dimensional palatal volume measurement represents the morphology of the palate in all planes. Intraoral scanning of patients or scanning dental casts are radiation-free three-dimensional imaging processes compared to computed tomography (CT) or cone beam computed tomography (CBCT) and are in recent interest for measuring palatal volume [21]. Studies investigating palatal volume have been performed in the past few years [8, 24]. Generali et al. [8] reported significantly smaller palatal volume for patients with unilateral cleft lip and palate compared to a non-cleft control at the mean age of 9.33 ± 1.67 years. Pucciarelli et al. [24] reported also significantly smaller palatal volume for patients with unilateral cleft lip and palate compared to a non-cleft control aged from 18 to 30 years. Three-dimensional imaging and palatal volume measurements have also been done for patients with mouth breathing and obstructive sleep apnea [13, 16]. Kecik [13] reported significantly smaller palatal volume for patients with obstructive sleep apnea compared to individuals without any symptom of obstructive sleep apnea. Lione et al. [16] reported also that changes in physiological upper airway function resulted in skeletal adaptions of the maxilla. The palatal volume was significantly smaller for patients with mouth breathing compared to nose breathing patients.

### Aims of the study

Since many different conclusions of craniofacial anomalies and their relation to the posterior airway space coexist, this comparative clinical study investigated the palatal morphology concerning volumetric size, posterior airway space dimension and the adenoids of patients with and without a cleft. The use of landmarks on three-dimensional intraoral scans and cephalometric radiographs should be verified as a probable method to analyze the palatal volume and dimension of the posterior airway space as well as the size of the area taken by the adenoids and maxillary position.

## Materials and methods

### Patients

All patients were exclusively diagnosed for orthodontic treatment at Saarland University Hospital between 2014 and 2024. All three-dimensional intraoral scans and cephalometric radiographs were chosen from pretreatment diagnostic records. Written informed consent was obtained from all patients or parents before using their three-dimensional intraoral scans and cephalometric radiographs for this study.

### Inclusion/Exclusion criteria

The presence of unilateral cleft lip and palate for group 1 (*n* = 15), bilateral cleft lip and palate for group 2 (*n* = 8) and non-cleft lip and palate for group 3 (*n* = 15) were the inclusion criteria. All patients showed transverse deficits of the maxilla. The patients with unilateral cleft lip and palate showed unilateral crossbites on the cleft side. The patients with bilateral cleft lip and palate showed bilateral crossbites. The non-cleft control patients showed also transverse deficits, but only four of them had unilateral crossbites, the other 11 patients showed no crossbite, but had a micrognathic maxilla. Exclusion criteria included comorbid syndromes, genetic disorders, Pierre Robin sequence and patients with an isolated cleft lip or palate.

As a precondition, diagnostic data including three-dimensional intraoral scans and digital cephalometric radiographs had to be present. Data were extracted from before the beginning of orthodontic treatment.

### Palatal volume and cephalometric measurement

A total of 38 three-dimensional intraoral scans and 38 cephalometric radiographs of patients with and without cleft lip and palate from one orthodontic clinic were available. A subdivision by gender was not performed. The three-dimensional intraoral scans were measured using the program MeshLab (Visual Computing Lab, Institute of Information Science and Technologies „Alessandro Faedo“ – National Resarch Council of Italy, Pisa, Italy) and the cephalometric radiographs were measured using the software OnyxCeph^®^ 3TM (Image Instruments GmbH, Chemnitz, Germany).

### Landmarks and measuring technique

**Fig. 1:**
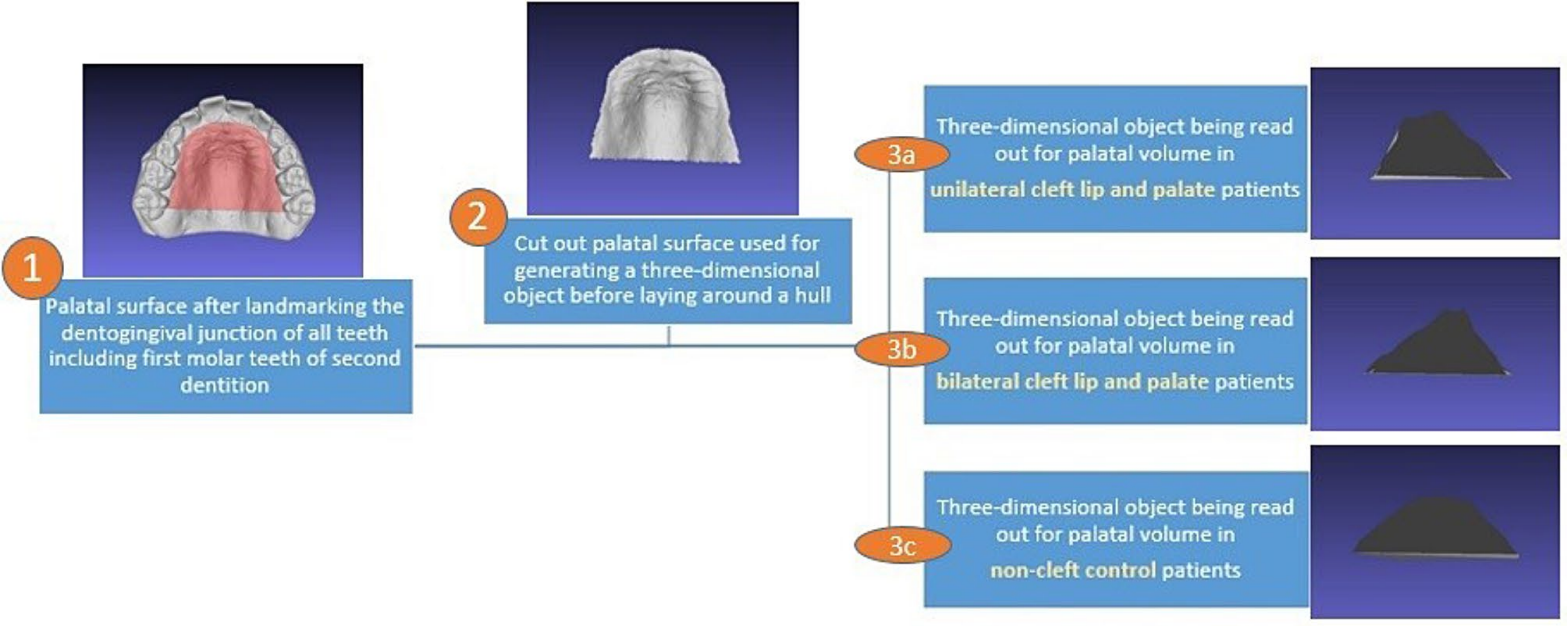
Workflow of palatal volume measurement of patients with and without cleft lip and palate

The technique for palatal volume evaluation of the three-dimensional intraoral scans has been predefined by us at the beginning of the study based on Türkyilmaz [28] and was used for each three-dimensional intraoral scan in the same way. The palatal surface has been landmarked at the dentogingival junction of all teeth including first permanent molars. The palatal surface was cut out afterwards. A hull was laid around the palatal surface and a three-dimensional object of the palatal area has been generated. After that, the volume of the new generated object was read out. (Fig. 1).

**Fig. 2:**
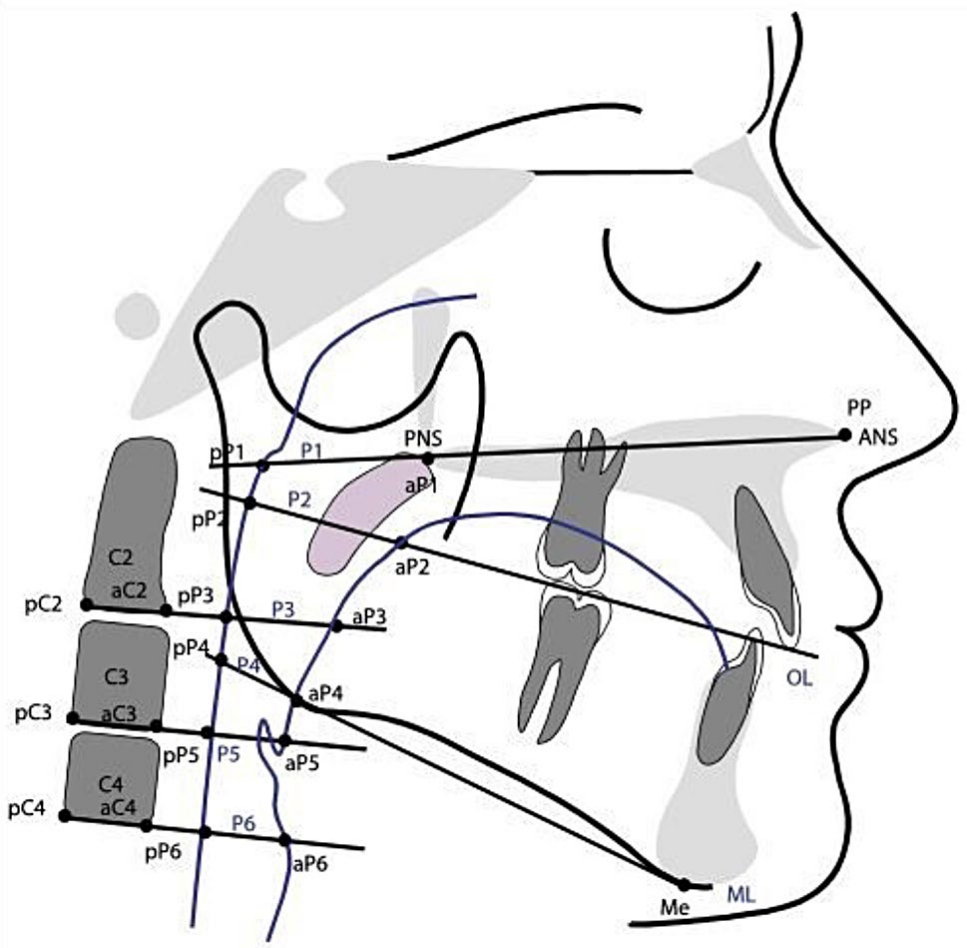
Overview of the landmarks used on the cephalometric radiographs and the linear and angular parameters calculated from them according to Kinzinger et al.

**Fig. 3:**
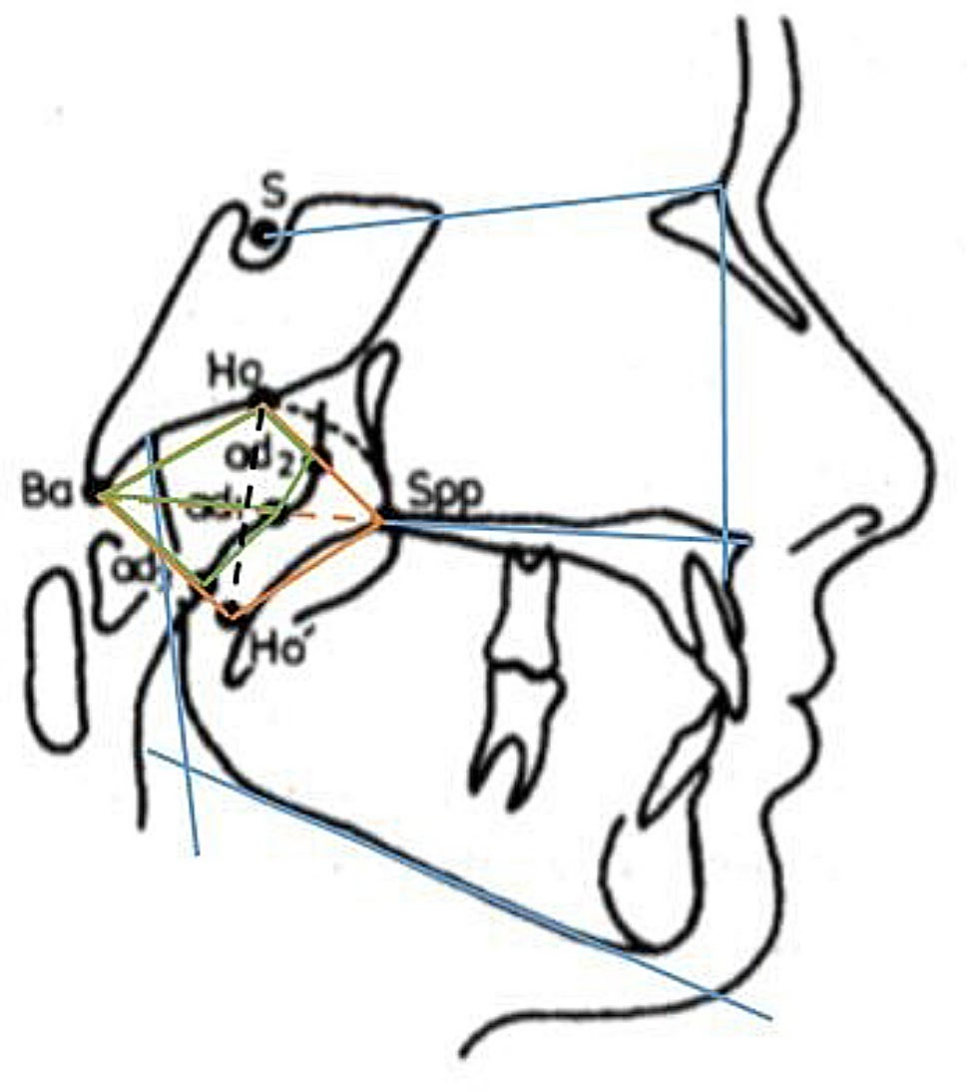
Overview of the landmarks used on the cephalometric radiographs and the linear and angular parameters calculated from them according to Jonas and Mann

The cephalometric radiograph evaluation was based on landmarks defined and used by Kinzinger et al. [14] and Jonas and Mann [11] for calculating distances, areas, dimensions, percentages and angles (Table 1) in all groups (Figs. 2 and 3).

**Table 1:** Cephalometric landmarks and measurements

*Measurement*	
**Distances (mm)**	
S-Ba	length of the clivus: distance between the central point of the sella turcica (Sella, (S)) and the most inferior posterior point of the anterior border of the foramen magnum (Basion, (Ba))
S-Spp	length of the posterior upper face height: distance between landmark Sella (S) and the most posterior point on the maxilla (Spina nasalis posterior, (Spp)/posterior nasal spine, (PNS))
Ba-Spp	depth of the bony nasopharynx: distance between landmark Basion (Ba) and Spina nasalis posterior (Spp)
P1	distance of the point of intersection of the nasal line and the posterior pharyngeal wall (posterior nasopharynx, (pP1)) and the point of intersection of the nasal line and the anterior pharyngeal wall (anterior nasopharynx (aP1); analog points: Spina nasalis posterior (Spp)/posterior nasal spine, (PNS))
P2	distance of the point of intersection of the occlusal plane and the posterior pharyngeal wall (superior posterior oropharynx, (pP2)) and the point of intersection of the occlusal plane and the anterior pharyngeal wall (superior anterior oropharynx, (aP2))
P3	distance of the point of intersection of the distance of the most anterior and posterior inferior point of the vertebral body C2 (aC2-pC2) and the posterior pharyngeal wall (inferior posterior oropharynx, (pP3)) and the point of intersection of the distance aC2-pC2 and the anterior pharyngeal wall (inferior anterior oropharynx, (aP3)) at the level of C2
P4	distance of the point of intersection of the mandibular line and the anterior pharyngeal wall (superior posterior laryngopharynx, (pP4)) and the point of intersection of the mandibular line and the anterior pharyngeal wall (superior anterior laryngopharynx, (aP4)) at the mandibular level
P5	distance of the point of intersection of the distance of the most anterior and posterior inferior point of the vertebral body C3 (aC3-pC3) and the posterior pharyngeal wall (inferior posterior laryngopharynx, (pP5)) and the point of intersection of the distance aC3-pC3 and the anterior pharyngeal wall (inferior anterior laryngopharynx, (aP5)) at the level of C3
P6	distance of the point of intersection of the distance of the most anterior and posterior inferior point of the vertebral body C4 (aC4-pC4) and the posterior pharyngeal wall (posterior subglottic area, (pP6)) and the point of intersection of the distance aC4-pC4 and the anterior pharyngeal wall (anterior subglottic area, (aP6)) at the level of C4
**Areas (mm** ^**2**^ **)**	
Spp-Ho-Ba-Spp	area of the bony nasopharynx: measured between the landmark Spina nasalis posterior (Spp), the most posterior intersection of the Os sphenoidale and the vomer (Hormion, (Ho)) and the landmark Basion (Ba)
Spp-Ho-Ba-Ho´-Spp	dimension of the entire nasopharynx: measured between the landmarks Spina nasalis posterior (Spp), Hormion (Ho), Basion (Ba) and the projection of Ho about the distance Ba-Spp (Hormion´, (Ho´))
ad2-Ho-Ba-ad1-ad2	dimension of the adenoids in the area of the bony nasopharynx: measured between the point of intersection of the line Ho-Spp and the posterior pharyngeal wall (ad2), the landmarks Hormion (Ho), Basion (Ba) and the point of intersection of the line Ba-Spp and the posterior pharyngeal wall (ad1)
ad2-Ho-Ba-ad3-ad2	overall dimension of the adenoids in the entire nasopharynx: measured between the landmarks ad2, Hormion (Ho), Basion (Ba) and the point of intersection of the line Ba-Ho´ and the posterior pharyngeal wall (ad3)
**Percentages (%)**	
ad2-Ho-Ba-ad1-ad2/Spp-Ho-Ba-Spp	adenoids in relation to the bony nasopharynx
ad2-Ho-Ba-ad3-ad2/Spp-Ho-Ba-Ho´-Spp	adenoids in relation to the entire nasopharynx
**Angles (°)**	
SNA	angle between the cranial base (SN) and the deepest point on the curvature of the anterior surface of the maxilla (Point A, (A))
NL/SN	angle between the distance Spa-Spp (nasal line, (NL)) and the cranial base (SN)
ML-NL	angle between the mandibular plane (ML) and the distance Spa-Spp (nasal line, (NL))
MeGoAr	gonial angle: angle between the most inferior point of the mandibular symphysis (Menton, (Me)), the most inferior posterior point of the mandibular angle (Gonion, (Go)) and the intersection of the dorsal contour of the condylar head and the contour of the posterior cranial base (Articulare, (Ar))

The clivus length, superior posterior face height, depth of the nasopharynx, posterior airway space at different levels, area of the bony nasopharynx, dimension of the entire nasopharynx and adenoids in the area of the bony and entire nasopharynx were evaluated and percentages of the adenoids in relation to the bony and the entire nasopharynx were measured.

The angles SNA, NL/SN, ML-NL, MeGoAr were used to evaluate the sagittal and vertical positions of maxilla and mandibula and the growth pattern.

### Statistical method, error of the method

Statistical analysis was performed with the SPSS software version 28 (IBM Corporation, Armonk, NY, USA). Statistics included Shapiro-Wilk-Test and simple ANOVA (Bonferroni) for the three-dimensional intraoral scans and cephalometric radiographs. The level of significance was set at *p* < 0.05. The significance level was defined as follows: *p* ≥ 0.05 not significant, *p* < 0.05 significant, *p* < 0.01 highly significant and *p* < 0.001 most highly significant. The effect size was tested by the formula f = √ƞ^2^/1- ƞ^2^ using Cohen´s criteria (for f): 0.10 = small effect size and low correlation, 0.25 = moderate effect size and correlation and 0.40 = large effect size and high correlation. For testing the intrarater-reliability the palatal volume measurement was repeated on all 38 three-dimensional intraoral scans of every patient with and without cleft lip and palate two months after the first investigation. Measuring accuracy was verified using Pearson correlation test. The correlation *r* was > 0.5 for palatal volume measurement. The correlation was strong and positive. For testing the intrarater-reliability of the cephalometric radiographs, the evaluation process was repeated on 30% of each group two months after the first investigation to evaluate the impact of landmarking errors, which involved removing and replacing the markings. The differences were statistically analyzed using Dahlberg´s error of the method (MF) with the formula MF = √(∑d^2^/2n), where *d* is the difference between two measurement results and *n* is the number of duplicate measurements [4]. The MF for angular and linear measurements in the present study was < 1 for all measurements. For testing the correlation *r* between the three-dimensional intraoral scans and cephalometric radiographs, again Pearson correlation test was used.

## Results

### Patients

The patients were divided into three groups (uni- or bilateral cleft lip and palate (UCLP, BCLP) and non-cleft control), and compared to each other. Three-dimensional intraoral scans and cephalometric radiographs of 38 non-syndromic patients (15 UCLP, 8 BCLP, 15 non-cleft control) at the age of 8.57 ± 1.79 years (UCLP), 8.46 ± 1.37 years (BCLP) and 9.03 ± 1.02 years (non-cleft control) were retrospectively identified and analyzed.

All patients with cleft lip and palate (*n* = 15 UCLP, *n* = 8 BCLP) were matched with a non-cleft control (*n* = 15). The control had no prior orthodontic treatment. Patients selected for control were referred by general dentists, and presented themselves for treatment of unilateral crossbites and/or skeletal class II, division 1 anomalies.

### Palatal volume (Table 2)

**Table 2:** Palatal volume [mm^3^] for UCLP, BCLP and non-cleft control. Pretreatment visit, *M* Mean, *SD* standard deviation, ^a^Simple ANOVA/Bonferroni between groups

	UCLP	BCLP	Non-cleft control	*P* value^a^
	M ± SD	M ± SD		
*Palatal Volume*	3020.85 ± 800.49	2294.08 ± 563.06	4471.02 ± 887.86	UCLP/non-cleft control: < 0.001
				BCLP/non-cleft control: < 0.001
				UCLP/BCLP: 0.134

For UCLP and BCLP patients the palatal volume was significantly smaller than for the non-cleft control (UCLP: 3020.85 ± 800.49 mm^3^; BCLP: 2294.08 ± 563.06 mm^3^; non-cleft control: 4471.02 ± 887.86 mm^3^; p = < 0.001; f = 1.144). The difference between UCLP and BCLP patients was not significant (*p* = 0.134).

### Cephalometric measurements

#### Bony structures (Table 3)

**Table 3:** Bony structures [mm] for UCLP, BCLP and non-cleft control. Pretreatment visit, *M* Mean, *SD* standard deviation, ^a^Simple ANOVA/Bonferroni between groups

	UCLP	BCLP	Non-cleft control	*P* value^a^
	M ± SD	M ± SD		
*Bony structures*				
Clivus length	37.04 ± 4.15	35.64 ± 2.15	38.13 ± 4.58	0.374
Superior posterior face height	36.04 ± 2.95	35.50 ± 2.08	42.65 ± 2.70	UCLP/non-cleft control: < 0.001
				BCLP/non-cleft control: < 0.001
				UCLP/BCLP: < 0.001
Depth of bony nasopharynx	38.79 ± 3.01	36.14 ± 3.21	40.57 ± 2.71	BCLP/non-cleft control: 0.004
				UCLP/BCLP: 0.140
				UCLP/non-cleft control: 0.317

In UCLP and BCLP patients the length of the clivus was smaller than in the non-cleft control (UCLP: 37.04 ± 4.15 mm; BCLP: 35.64 ± 2.15 mm; non-cleft control: 38.13 ± 4.58 mm; *p* = 0.374).

In UCLP and BCLP patients the superior posterior face height was significantly smaller than in the non-cleft control (UCLP: 36.04 ± 2.95 mm; BCLP: 35.50 ± 2.08 mm; non-cleft control: 42.65 ± 2.70 mm; p = < 0.001; f = 1.288). In BCLP patients the depth of the bony nasopharynx was significantly smaller than for non-cleft control (UCLP: 38.79 ± 3.01 mm; BCLP: 36.14 ± 3.21 mm; non-cleft control: 40.57 ± 2.71 mm; *p* = 0.004; f = 0.584). The differences between UCLP and BCLP patients and UCLP patients and the non-cleft control were not significant (*p* = 0.140 and *p* = 0.317).

### Posterior airway space depth (Table 4)

**Table 4:** Posterior airway space depth [mm] for UCLP, BCLP and non-cleft control. Pretreatment visit, *M* Mean, *SD* standard deviation, ^a^Simple ANOVA between groups

	UCLP	BCLP	Non-cleft control	*P* value^a^
	M ± SD	M ± SD		
*Posterior airway space*				
Palatal level	13.75 ± 3.81	10.56 ± 3.48	14.25 ± 3.83	0.08
Occlusal plane level	7.65 ± 2.40	8.66 ± 1.99	9.03 ± 2.01	0.223
C2 level	8.53 ± 2.97	7.65 ± 2.48	10.10 ± 2.45	0.095
Mandibular level	9.39 ± 3.35	8.91 ± 3.11	10.93 ± 2.63	0.236
C3 level	7.69 ± 3.22	8.35 ± 3.15	8.83 ± 2.90	0.602
C4 level	9.61 ± 3.27	10.60 ± 4.19	11.38 ± 2.00	0.297

In BCLP patients the depth of the posterior airway space at the palatal level was smaller than in UCLP patients and the non-cleft control (UCLP: 13.75 ± 3.81 mm; BCLP: 10.56 ± 3.48 mm; non-cleft control: 14.25 ± 3.83 mm; *p* = 0.080). In UCLP patients the depth of the posterior airway space at the occlusal plane level was smaller than in BCLP patients and the non-cleft control (UCLP: 7.65 ± 2.40 mm; BCLP: 8.66 ± 1.99 mm; non-cleft control: 9.03 ± 2.01 mm; *p* = 0.223). In BCLP patients the depth of the posterior airway space at the level of C2 was smaller than in UCLP patients and the non-cleft control (UCLP: 8.53 ± 2.97 mm; BCLP: 7.65 ± 2.48 mm; non-cleft control: 10.10 ± 2.45 mm; *p* = 0.095). In BCLP patients the depth of the posterior airway space at the mandibular level was smaller than in UCLP patients and the non-cleft control (UCLP: 9.39 ± 3.35 mm; BCLP: 8.91 ± 3.11 mm; non-cleft control: 10.93 ± 2.63 mm; *p* = 0.236). In UCLP patients the depth of the posterior airway space at the level of C3 was smaller than in BCLP patients and the non-cleft control (UCLP: 7.69 ± 3.22 mm; BCLP: 8.35 ± 3.15 mm; non-cleft control: 8.83 ± 2.90 mm; *p* = 0.602). In UCLP patients the depth of the posterior airway space at the level of C4 was smaller than in BCLP patients and the non-cleft control (UCLP: 9.61 ± 3.27 mm; BCLP: 10.60 ± 4.19 mm; non-cleft control: 11.38 ± 2.00 mm; *p* = 0.297).

### Nasopharynx areas and adenoids size (Table 5)

**Table 5:** Nasopharynx areas and adenoids size [mm^2^] for UCLP, BCLP and non-cleft control. Pretreatment visit, *M* Mean, *SD* standard deviation, ^a^Simple ANOVA between groups

	UCLP	BCLP	Non-cleft control	*P* value^a^
	M ± SD	M ± SD		
*Nasopharynx areas*				
Area of bony nasopharynx	349.56 ± 71.78	363.03 ± 62.72	384.81 ± 72.28	0.395
Dimension entire nasopharynx	699.11 ± 143.58	726.05 ± 125.45	769.63 ± 144.55	0.395
*Adenoids size*				
Adenoids in area of bony nasopharynx	240.07 ± 63.14	248.79 ± 58.39	244.07 ± 66.80	0.951
Adenoids in entire nasopharynx	382.43 ± 82.42	378.73 ± 77.95	318.80 ± 84.99	0.089

In UCLP patients the area of the bony nasopharynx was smaller than in BCLP patients and the non-cleft control (UCLP: 349.56 ± 71.78 mm^2^; BCLP: 363.03 ± 62.72 mm^2^; non-cleft control: 384.81 ± 72.28 mm^2^; *p* = 0.395). In UCLP patients the dimension of the entire nasopharynx was smaller than in BCLP patients and the non-cleft control (UCLP: 699.11 ± 143.58 mm^2^; BCLP: 726.05 ± 125.45 mm^2^; non-cleft control: 769.63 ± 144.55 mm^2^; *p* = 0.395). In BCLP patients the dimension of the adenoids in the area of the bony nasopharynx was bigger than in UCLP patients and the non-cleft control (UCLP: 240.07 ± 63.14 mm^2^; BCLP: 248.79 ± 58.39 mm^2^; non-cleft control: 244.07 ± 66.80 mm^2^; *p* = 0.951). In UCLP patients the overall dimension of the adenoids in the entire nasopharynx was bigger than in BCLP patients and the non-cleft control (UCLP: 382.43 ± 82.42 mm^2^; BCLP: 378.73 ± 77.95 mm^2^; non-cleft control: 318.80 ± 84.99 mm^2^; *p* = 0.089).

### Adenoids percentages (Table 6)

**Table 6:** Adenoids percentages [%] for UCLP, BCLP and non-cleft control. Pretreatment visit, *M* Mean, *SD* standard deviation, ^a^Simple ANOVA/Bonferroni between groups

	UCLP	BCLP	Non-cleft control	*P* value^a^
	M ± SD	M ± SD		
*Adenoids Percentage*				
Adenoids/bony nasopharynx	68.34 ± 8.86	68.08 ± 6.82	62.22 ± 8.10	0.099
Adenoids/entire nasopharynx	55.75 ± 11.39	52.16 ± 4.95	41.58 ± 8.99	UCLP/non-cleft control: 0.001
				BCLP/non-cleft control: 0.049
				UCLP/BCLP: 0.688

In UCLP patients the percentage of the adenoids in relation to the bony nasopharynx was bigger than in BCLP patients and the non-cleft control (UCLP: 68.34 ± 8.86%; BCLP: 68.08 ± 6.82%; non-cleft control: 62.22 ± 8.10%; *p* = 0.099). In UCLP patients the percentage of the adenoids in relation to the entire nasopharynx was significantly bigger than in the non-cleft control (UCLP: 55.75 ± 11.39%; BCLP: 52.16 ± 4.95%; non-cleft control: 41.58 ± 8.99%; *p* = 0.001; f = 0.730). In BCLP patients the percentage of the adenoids in relation to the entire nasopharynx was significantly bigger than in the non-cleft control (*p* = 0.049; f = 0.730). The difference between UCLP and BCLP patients was not significant (*p* = 0.688).

### Angles (Table 7)

**Table 7:** Angles [°] for UCLP, BCLP and non-cleft control. Pretreatment visit, *M* Mean, *SD* standard deviation, ^a^Simple ANOVA/Bonferroni between groups

	UCLP	BCLP	Non-cleft control	*P* value^a^
	M ± SD	M ± SD		
*Angles*				
SNA	79.37 ± 4.23	80.78 ± 4.30	82.32 ± 2.69	0.107
NL/SN	13.13 ± 4.17	13.91 ± 1.89	6.65 ± 3.63	UCLP/non-cleft control: < 0.001
				BCLP/non-cleft control: < 0.001
				UCLP/BCLP: 0.885
ML-NL	22.95 ± 6.04	24.81 ± 6.06	25.18 ± 5.36	0.548
MeGoAr	128.35 ± 6.78	129.80 ± 3.43	126.24 ± 6.59	0.393

In UCLP patients the angle SNA was smaller than in BCLP patients and the non-cleft control (UCLP: 79.37 ± 4.23°; BCLP: 80.78 ± 4.30°; non-cleft control: 82.32 ± 2.69°; *p* = 0.107).

In UCLP and BCLP patients the angle NL/SN was significantly bigger than in the non-cleft control (UCLP: 13.13 ± 4.17°; BCLP: 13.91 ± 1.89°; non-cleft control: 6.65 ± 3.63°; p = < 0.001; f = 0.961). The difference between UCLP and BCLP patients was not significant (*p* = 0.885).

In UCLP patients the angle ML-NL was smaller than in BCLP patients and the non-cleft control (UCLP: 22.95 ± 6.04°; BCLP: 24.81 ± 6.06°; non-cleft control: 25.18 ± 5.36°; *p* = 0.548). In UCLP and BCLP patients the angle MeGoAr was bigger than in the non-cleft control (UCLP: 128.35 ± 6.78°; BCLP: 129.80 ± 3.43°; non-cleft control: 126.24 ± 6.59°; *p* = 0.393).

### Pearson correlation for three-dimensional intraoral scans and cephalometric radiographs

In patients with unilateral cleft lip and palate there was a significant positive correlation between the overall dimension of the adenoids in the entire nasopharynx and palatal volume (*p* = 0.039; *r* = 0.536). There was also a significant positive correlation between the overall dimension of the adenoids in the entire nasopharynx and the superior posterior face height (*p* = 0.031; *r* = 0.557).

In patients with bilateral cleft lip and palate there was a significant positive correlation between the overall dimension of the adenoids in the entire nasopharynx and the depth of the bony nasopharynx (*p* = 0.002; *r* = 0.902).

In patients without a cleft there was a significant positive correlation between the overall dimension of the adenoids in the entire nasopharynx and the depth of the bony nasopharynx (*p* = 0.042; *r* = 0.530).

## Discussion

This study aimed to evaluate the differences of the palatal morphology, upper airway and adenoids between patients with uni- or bilateral cleft lip and palate and patients without a cleft. Because of the nature of the cleft and thereby the greater amount of surgery for space closure, patients with bilateral cleft lip and palate present more scar tissue than patients with unilateral cleft lip and palate. Therefrom, growth restrictions – especially in transverse and sagittal direction – tend to be more pronounced in patients with bilateral cleft lip and palate. In our study, the relevant differences between unilateral and bilateral cleft lip and palate patients were smaller palatal volume, clivus length, superior posterior face height, depth of bony nasopharynx and posterior airway space especially at palatal level in patients with bilateral cleft lip and palate underlining the growth restrictions.

Since further growth in patients with a cleft is always accompanied by growth restrictions, the differences between patients with bilateral and unilateral cleft lip and palate as described before appear more pronounced in older patients compared to the younger patients of our study, particularly when left untreated. Reduced palatal volume due to growth restrictions is often associated with anterior and/or lateral crossbites and should be treated early in age. Advancement of maxillary growth in sagittal and/or transverse direction by means of protraction and/or expansion of the maxilla for crossbite correction, results in greater palatal volume. In addition, greater palatal volume has a positive impact on the nasal volume and the posterior airway space making nasal breathing easier. If growth is completed in older patients and crossbites persist, treatment of growth restrictions requires orthognathic surgery.

Since cephalometric radiographs are a useful diagnostic tool for evaluation craniofacial structures in sagittal and vertical direction, palatal volume measurement is helpful to consider the overall size of the palate, including the transverse. Patients with transverse deficiency of the maxilla showed smaller palatal volume. Furthermore, association exists between small palatal volume and small superior posterior face height and depth of the bony nasopharynx. The smaller the palatal volume, the bigger the percentage of the adenoids in relation to the entire nasopharynx. The bigger angle NL/SN in patients with a cleft lip and palate is explicable by posterior rotation of the maxilla due to scar tissue because of surgical closure of the cleft area [17, 19]. Only few studies are existing to evaluate dimensions of the upper airway in all three planes, because indications for CT/CBCT imaging are strictly predefined. For orthodontic and orthopedic purposes, cephalometric radiographs are still standard [27]. Karia et al. [12] compared posterior airway spaces of 39 patients with unilateral, 17 patients with bilateral cleft lip and palate, 7 patients with cleft palate and 42 patients without a cleft using CBCT images. Anteroposterior dimensions of the airway at the level of the postnasal spine, base of the tongue and epiglottis and height and volume of the oropharyngeal airway were significantly smaller in patients with a cleft compared to the non-cleft control. Eslami et al. [7] compared nasopharyngeal airway volume of 14 patients with unilateral, 10 bilateral cleft lip and palate and 16 patients without a cleft using CBCT images. They also reported significant differences between patients with and without a cleft. Middle pharyngeal volume and nasal width was significantly smaller for patients with a cleft. Miranda-Viana et al. [20] compared 298 CBCT images of non-cleft 144 males and 154 females with different skeletal malocclusions, facial types and breathing patterns. They associated a greater height of the hard palate with a lower volume of the upper airways and a greater width of the hard palate with a higher volume of the upper airways. They also observed an association between the width and height of the hard palate at the first molars region and the total volume of the maxillary sinuses. De Oliveira et al. [5] compared craniofacial and dental arch morphology and pharyngeal airway space of 108 non-cleft adolescents aged between 12 and 17 years using cephalometric radiographs. Their findings suggested gender-dependent correlations of the nasopharyngeal and oropharyngeal airway space with the sagittal craniofacial morphology and the transverse dental arch form. The length of the maxilla was directly proportional to the upper nasopharyngeal airway dimensions in males and females. In females, the upper arch form appeared to be related to oropharyngeal measurements.

### Limitations

Three-dimensional intraoral scans of patients of University Hospital and Dental Medical School Saarland with and without cleft lip and palate were analyzed, excluding patients with comorbid syndromes, genetic disorders, Pierre robin sequence or isolated cleft lip or palate. Due to the small number of patients, a gender division was not performed. The number of patients per group remained low, because patients with a cleft lip and palate, especially those with bilateral cleft lip and palate are rare. Out of 20 patients with unilateral and 12 patients with bilateral cleft lip and palate, only 15 patients with unilateral and 8 patients with bilateral cleft lip and palate were appropriate participants for this study due to strict inclusion/exclusion criteria. However, division into different groups was mandatory for comparison of patients with different pathologies. Furthermore, only few studies of patients with bilateral cleft lip and palate exist. Comparison with plaster models or three-dimensional intraoral scans from other centers to increase numbers was not used due to different treatment protocols. The age-matched non-cleft control patients presented themselves for orthodontic treatment as indicated by a general practitioner, mainly because of crossbites. Therefore, suitability of the control is only partially given, but it was possible to gain evidence about palatal volume, upper airway dimension and adenoids in an age-matched control. It was even possible to judge skeletal parameters requiring a radiographic comparison. At that point of examination cephalometric radiographs were indicated. The study was restricted to growing patients prior to orthodontic treatment, because the morphology of the palate and the whole maxilla changes due to orthodontic appliances. The volume of the posterior airway space varies depending on the respiratory cycle influencing the measurements of the cephalometric radiographs. Instructing patients to hold their breath during the X-ray can solve this problem. Cephalometric radiographs are helpful for screening, but for diagnosis of airway obstruction further diagnostic techniques are mandatory.

It should be acknowledged, that a single examiner conducted the investigation. Therefore, a degree of subjectivity may exist despite time-shifted intrarater reliability control.

## Conclusion

Patients with cleft lip and palate have a significantly smaller palatal volume due to different palatal morphology compared to patients without a cleft.

The morphology of the palate and especially transverse deficiency of the maxilla relates to the upper airway. Even the adenoids seem to be affected, especially for cleft lip and palate patients.

## Data Availability

No datasets were generated or analysed during the current study.
